# The family situation of wards under the supervision of court - appointed custodians during COVID-19 pandemic in Poland: a self-report study to examine the perception of probation officers.

**DOI:** 10.12688/f1000research.110435.1

**Published:** 2022-12-19

**Authors:** Robert Opora

**Affiliations:** 1 Department of of Social Sciences, University of Gdansk, Bażyńskiego 4, Gdańsk, 80-309, Poland

**Keywords:** Pandemic COVID – 19, Social problems, Probation supervision, family, social rehabilitation

## Abstract

Background: The Polish Probation Service is strongly linked to social work. This text presents how the work and the problems of clients have changed in the Polish Probation Service during the COVID-19 pandemic. The article presents empirical data. Methods: A questionnaire was used among 216 Polish probation officers. Results: The obtained results describe the changes in the problems experienced by clients of the Probation Service during the COVID-19 pandemic. Conclusions: Since we can expect further increase in the frequency of occurring personal and social problems experienced by court-appointed custodians’ wards, financial resources and energy of social institutions, cooperation with court guardianship should be focused on psycho-educational campaigns concentrating on providing information on symptoms of problems in the mental and emotional sphere. Preventing exclusion of groups at risk in the labour market and supporting families to prevent the escalation of psychopathology, development of addictions and domestic violence should be priority areas.

## Introduction

Although we do not yet know all of the consequences of the COVID-19 pandemic, after over a year we can begin to describe the reality we find ourselves in. The COVID-19 pandemic was a global issue. Related experiences affected every person irrespective of their country and community and forced us to change our lifestyles and ways of working. People have faced pressures in restricting social interactions, especially with their relatives, due to the necessity of limiting virus transmission.

The Polish Probation Service is strongly linked to social work. Similarly to all social and professional groups, during the pandemic the Probation Service had to adjust to limitations concerning public health. This entailed limitations on physical contact and increased dependency on remote supervision.

Simultaneously, wards, especially those with the most complex needs, experienced a decrease in emotional wellbeing which worsened as a result of the isolation and limited access to institutional forms of support ( Carr, 2021). The area of interpersonal relations which usually supports people in difficult situations, was limited. It is of a particular significance especially for persons who do not easily establish social relations, and for whom workplace or school and participation in out-of-school activities provided space in which these relations can be established.

Social distancing has made it difficult to maintain relationships and a growing feeling of loneliness. Pandemic-related fear and uncertainty has a negative impact on the family system. People may fear for the health and life of their grandparents or parents ( Ptaszek *et al.*, 2020). Furthermore, the restrictions and related isolation may reinforce or disclose family dysfunctions.

In many European countries, probation is an important element of the social work system. Hence, the concept of social work as work with people who violate social norms is common ( Vanstone, 2012). The tasks of probation officers include enabling their clients who violate social norms to overcome difficult life situations using their own resources and abilities. The probation officers perform their work with families experiencing various social issues such as bad economic situations, unemployment, addiction, breakdown of social and family relations or violation of legal orders ( Janus-Dębska and Gronkiewicz-Ostaszewska, 2019). The ward’s family situation is influenced by the quality and quantity of activities undertaken by a court-appointed custodian. Limitations resulting from the restrictions introduced during the pandemic affected various spheres of human functioning.

Due to social distancing, closure of workplaces, remote work and introduction of distanced teaching, families faced new challenges. In case of some families or particular members thereof these circumstances contributed to the occurrence of a crisis or reinforced existing ones. In response to these challenges, the institution of probation has adapted, struggled, and ultimately endured to continue serving the court and the community.

Describing the reality of each individual area of Poland which experienced the pandemic is not possible because of the limited research. This study aims to capture generally what practice looked like pre-pandemic and how the discipline responded when confronted by this novel challenge. As terms like “social distancing,” and “quarantine” were added to the country’s lexicon, the probation officers raced to keep the courts running, rights preserved, and communities safe. Some of the responsibilities of the probation officers were possible to run via video conferences. However, as anyone who has used video conferencing software is aware, virtual communication is rarely a substitute for in-person interaction. While some may determine that the benefits of not having to travel outweigh the costs of having an online meeting, we do not, and cannot, find a balance with client rights and support ( Whaley *et al.*, 2021). Assessment interviews are now completed by videoconferencing or over the phone. Neither of these options is ideal; quickly building rapport with a client is much more difficult in a virtual environment. However, videoconferencing does offer some parallels to being in person. Being able to see one another allows both parties to give and receive non-verbal feedback such as nodding or leaning in, it assists in distinguishing between whether the speaker is taking a thoughtful pause or has finished speaking. Although videoconferencing is significantly different from in-person communication, a probation officer can create a trusting and comfortable environment for the defendant over video. Unfortunately, doing so becomes difficult when interviews are conducted over the phone. There is no longer body language or non-verbal feedback; accidental interruptions become more frequent; and a sense of connection is even more difficult to foster. The pandemic posed significant complications in the way officers carried out their duties.

**Figure 1.**
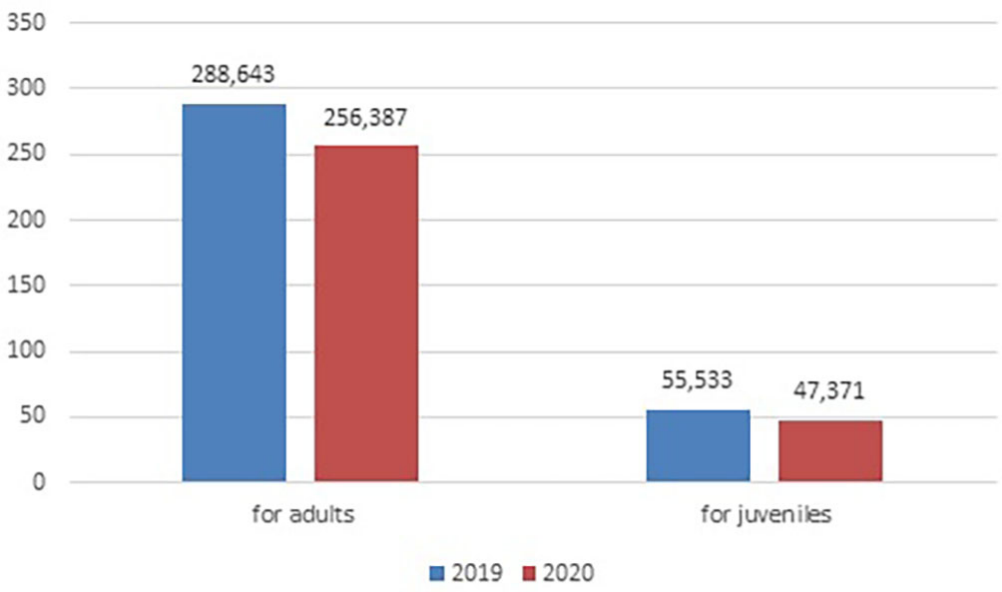
Comparation of new cases in the probation system reported between 2019 and 2020. Source: Own analyzes based on the statistics of the Ministry of Justice, Department of Strategy and European Funds, MS-S40r Report on the activity of the probation court service for the year 2019, 2020. Warszawa.

**Figure 2.**
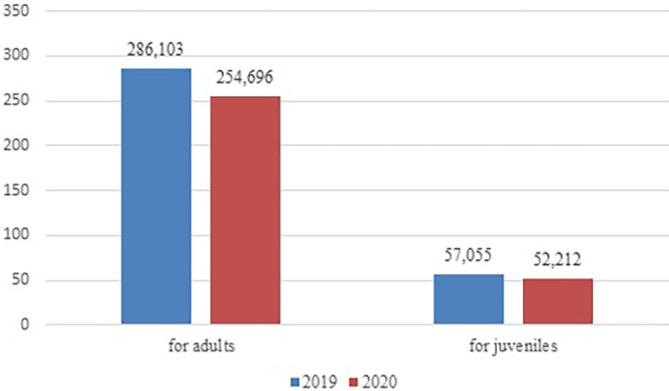
Comparation of completed cases in the probation system between 2019 and 2020. Source: Own analyzes based on the statistics of the Ministry of Justice, Department of Strategy and European Funds, MS-S40r Report on the activity of the probation court service for the year 2019, 2020. Warszawa.

As we see in Figures 1 and 2, both for adult and juvenile probation officers in 2022, compared to 2019, the number of new cases reported and completed cases decreased. Whether the decrease in adjudications was a direct result of reduced procedural efforts (stemming from a shift in investigation and charging practices by the executive branch and other agencies) or limitations on in-person court proceedings related to the pandemic is unclear.

The impact of a reduced caseload and the constraints posed by the pandemic for many probation officers did not mean a decrease in work. The pandemic made their work more complex because access to most social services became limited and probation officers had to look for alternative interventions for their clients. Unsurprisingly, officers used the temporary decrease in caseload to take on additional duties, and to assist their clients more because a lot of other social agencies closed themselves at the beginning of the pandemic.

The aim of the research was to learn opinions of probation officers regarding changes in the scope of social problems encountered by them in their performance of tasks during the pandemic. Thus, the following research question was asked:

Have social problems in families under the supervision of a court-appointed custodian intensified during the pandemic?

## Methods

### Study design

The research was conducted in 12 randomly selected court regions in Poland among 216 professional court-appointed custodians. The questionnaire was sent to all probation officers from these court regions. A total of 482 probation officers received the questionnaire, out of which 216 returned them. All of them were completed because the system didn’t allow uncompleted questionnaires to be returned.

The study was of a quantitative nature and was conducted using a diagnostic anonymous survey developed for the purposes of the research. The link with a survey questionnaire was developed in Microsoft Forms and shared by the University of Gdańsk with the chosen offices of District Probation Officers in an electronic form. Then the questionnaires were sent by e-mail by the office of District Probation Officers to all probation officers from the district. The probation officers were asked to fill in the questionnaire by using the link attached to the email with the instructions. The research began on August 01, 2021 and lasted one month until August 30, 2021. The research was anonymous and voluntary. A copy of the questionnaire can be found under *Extended data* ( Opora, 2022).

### Study population

The research was conducted in 12 randomly selected court regions in Poland among 216 of 480 professional court-appointed custodians. To be eligible for the study, respondents had to be a professional probation officer. District courts participating in the study were randomly selected from 46 districts in Poland. A potential source of bias in the research was the respondents’ limited work experience. They had no more than seven years of work experience in the probation system.

### Ethics and consent

On 13 July 2021, the study received the approval of the Ethics Committee of the Faculty of Psychology of the Gdansk University, Poland (approval no. 20/2021). Participants gave written informed consent for the use and publication of their data.

### Data collection

The research was conducted in August 2021, that is, after approximately 1.5 years since the announcement of the pandemic in Poland. The survey was created and completed using the MS Forms platform. The link was sent out through e-mail by the secretariats of the district courts to all family probations officers from the chosen districts.

Before the final research, a pilot study was conducted among 12 custodians representing both criminal as well as family and juvenile teams. As a result of the obtained information and undertaken activities, the survey questionnaire was partially modified and adjusted. In the metric of the questionnaire, an additional question about occupational rank was introduced and a question about drug abuse was added. The final version of the questionnaire included a metric on basic demographic information such as gender and length of employment as a family probation officer and the main part of the questionnaire consisted of 16 questions to measure the variable defined as the family situation of probationers’ wards. Respondents were allowed to reply to each question by selecting one answer from four. The answers were as follows: “it was not before and it is not now”, “it was less before than now”, “it was the same before as now”, “it was more before than now”. In addition, the text was stylistically corrected.

At the beginning of the survey, questionnaire instructions informed respondents of the purposes and rules of conducting research. Furthermore, respondents were informed of the email address of the person responsible for the research implementation. At the beginning of the survey questionnaire, a request for consent to participate in the research was included. The respondent could at any time stop his or her participation by closing the website and not sending the results.

### Data analysis

The statistical analysis was conducted through Statistica software. Basic descriptive statistics were primarily used and, in order to determine statistical significance of differences in obtained frequency of answers to questions, chi2 test was used. The reliability of the final version of the questionnaire was tested using Cronbach’s Alpha 0.89 which indicated high internal consistency. The number of potentially eligible probation officers in the 12 selected districts was 480. The number of confirmed eligible probation officers was 480, and the number included in the study who completed questionnaires was 216.

Since the research was conducted online, only completed questionnaires could be submitted. Therefore, there was no problem regarding lack of data or incomplete data in collected answers. There was just one summary measure over time.

## Results

In the research undertaken, the majority of the respondents were women (70%), while men made up 30%.

In order to determine problems experienced by wards during the pandemic, custodians gave answers by comparing their current work experiences to experiences from before the pandemic. The full dataset can be found under *Underlying Data* ( Opora, 2022).

As shown in the Table 1, 55% of custodians participating in the research, the number of difficulties related to wards staying with demoralised persons during the pandemic has not changed since before the pandemic. However, according to 40.27% of custodians, these difficulties intensified during the pandemic. Some persons, especially students, at the time of introducing remote education were probably at a disposal of significantly more time and remained outside of the control of a school or parents. Among the respondents, 2.77% custodians stated that currently they have more difficulties with regard to their wards staying with demoralised persons. The test of statistical significance indicates an existing difference, which means that during the pandemic, despite recommendations regarding social distance, a number of problematic behaviours resulting from leaving juveniles with demoralised peers increased (chi ^2^=96.93; df=2; p<0.05).

**Table 1.** Wards’ family situation.

Wards’ situation	The problem did not occur earlier and it has not occurred currently (%)	The problem occurred to a smaller extent than currently (%)	The problem occurred to the same extent as currently (%)	The problem occurred to a larger extent than currently (%)	Total	Total %
Difficulties with regard to wards staying with demoralised persons	1.38	40.27	55.55	2.77	216	100%
Deteriorating financial situation of families under supervision/custody during the pandemic	4.16	54.62	38.42	2.77	216	100%
Family fights	1.38	67.12	31.01	0.46	216	100%
Alcohol abuse by wards’ family members	1.38	45.83	52.31	0.46	216	100%
Alcohol abuse by wards	0.92	44.44	53.24	1.38	216	100%
Narcotics/designer drugs use	3.24	35.64	58.33	2.77	216	100%
Domestic violence	1.85	56.94	39.35	1.85	216	100%
Problems with participation in classes (not logging in classes, inactivity)	10.18	74.53	13.42	1.85	216	100%
Professional passiveness	3.7	55.55	38.88	1.85	216	100%
Reporting complaints by the family to the custodian due to the improper behaviour of the ward	3.7	37.5	56.48	2.31	216	100%
Referring to educational-care and resocialization facilities/revoking custody due to the non-fulfilment of obligations imposed on the ward	8.79	26.85	59.72	4.62	216	100%
Aggressive behaviours of the ward towards household members	2.31	49.07	46.75	1.85	216	100%
Hating others	12.5	41.66	43.98	1.85	216	100%
Risky way of spending free time	5.55	40.27	49.07	5.09	216	100%
Aggressive behaviours of the ward towards friends	8.33	33.33	56.01	2.31	216	100%
Mental overload of the ward’s relatives	3.24	69.44	25.04	2.31	216	100%

Every second respondent (54.62%) stated that as a result of the pandemic they observed the deteriorating financial situation of families under supervision/custody. Whereas 2.77% respondents indicated that during the pandemic the situation of families under their care improved. Other respondents stated that the financial situation of families under supervision/custody from before the pandemic and during the pandemic has not changed. The distribution of this frequency indicates statistically significant differences (chi ^2^= 95.16; df=2; p<0.05) and allows us to draw conclusions on the deteriorating financial situation of custodians’ wards.

In the opinion of 67.12% of custodians participating in the research, the number of conflicts in families increased. Only 0.45% noticed a drop in this area during the pandemic. Other respondents did not indicate any change before pandemic and during the pandemic in this scope. The changes indicate differences in the distribution of given answers (chi ^2^=146.37; df=2; p<0.05), which proves the increase in conflicts among families under supervision or custody.

In the opinions of 45.83% of court-appointed custodians, alcohol abuse by wards’ family members increased. Only 0.46% of them claim that the abuse decreased and 52.31% believe that this problem remains at the same level. These results indicate the presence of an alcohol problem in families with regard to whom a court-appointed custodian was adjudicated. This problem has been additionally growing under the circumstances resulting from the limitations introduced due to the pandemic. The test of statistical significance indicates a significant difference in the scope of given answers (chi ^2^=104.90; df=2; p<0.05).

On the grounds of obtained results, it can also be concluded that the problem of alcohol abuse by wards increased. Such an opinion was given by 44.44% of court-appointed custodians. According to only 1.38% of respondents, this problem has decreased during the pandemic. Whereas 53.24% of respondents believe that this problem has already existed before the pandemic and continues to exist at the same level. Changes in the distribution of given answers are statistically significant (chi ^2^=100.72; df=2; p<0.05) and indicate the growing problem of alcohol abuse during the pandemic by persons with regard to whom a supervision of a court-appointed custodian has been adjudicated.

35.64% of court-appointed custodians are of the opinion that the use of narcotics and designer drugs has also increased. Only 2.77% believe that the situation improved during the pandemic and 58.33% state that they encounter this problem during the pandemic as often as before the pandemic. Differences in the distribution of given answers are statistically significant chi ^2^=104.51; df=2; p<0.05, which indicates that, in the court-appointed custodians’ opinion, the pandemic period intensified the problems of using narcotics and designer drugs.

As many as 74.53% of court-appointed custodians believe that the problem with not participating in school classes or inactivity during classes, increased. Only 1.85% of custodians believe that this problem decreased during the pandemic and 13.42% stated that this problem remained at the same level. These results indicate significant differences in the distribution of given answers (chi ^2^=220.09; df=2; p<0.05).

55.55% of court-appointed custodians observed while performing supervisions and custodies a growing professional passivity in families, 38.88% of custodians notice existence of this problem and believe that it currently remains at the same level as before the pandemic. Only 1.85% of respondents believe that this problem decreased during the pandemic. Thus undoubtedly, during the pandemic court-appointed custodians have observed a growing professional passiveness in families under supervisions or custodies (chi ^2^=101.69; df=2; p<0.05).

During the pandemic every third respondent (37.5%) has observed a growing frequency of reporting complaints by the family to the custodian due to the improper behaviour of the ward and 56.48% have noticed that this problem is at the same level during the pandemic as it was in the period before the pandemic. Only 2.31% of custodians responded that the number of these complaints is currently smaller. The obtained results indicate the growing number of problems during the pandemic engaging their attention and time. Differences in the distribution are statistically significant (chi ^2^=101.66; df=2; p<0.05).

26.85% of respondents perceive the period of the pandemic as the one in which referring to educational-care and resocialization facilities or revoking custody due to the non-fulfilment of obligations imposed on the ward are more frequent. 4.62% of studied custodians do not notice this trend and believe that the number of such interventions dropped. While 59.72% of respondents believe that interventions are as frequent as before the pandemic. Differences in the obtained distribution are statistically significant (chi ^2^=109.17; df=2; p<0.05).

**Figure 3.**
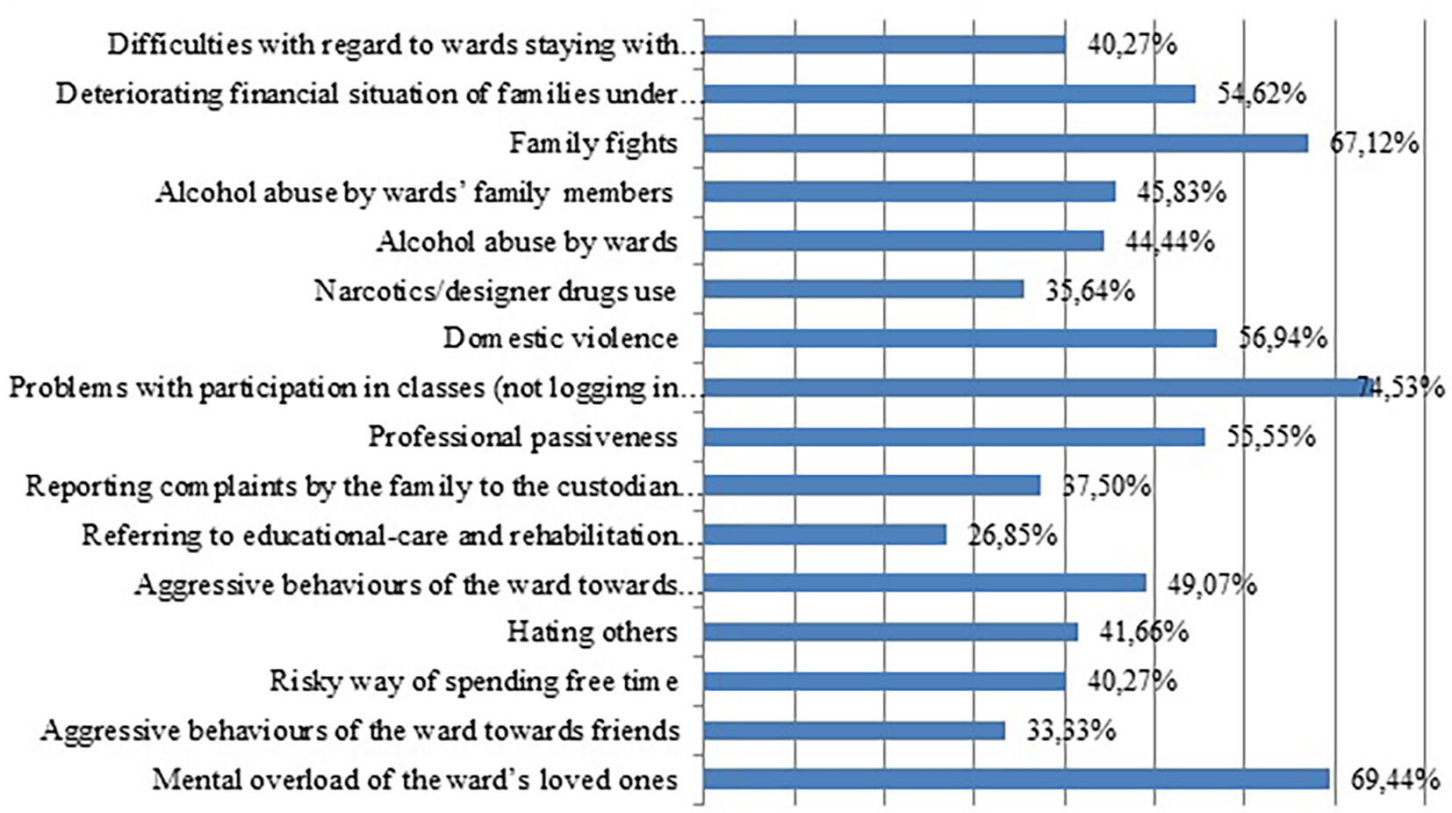
The increase in issues among families of wards under the court-appointed custodian’s supervision/custody during the pandemic.

As Figure 3 ilustrates, during the pandemic court-appointed custodians have noticed a significant growth in domestic violence (chi ^2^=104.56; df=2; p<0.05). Every second respondent (56.94%) agrees that they have more often encountered this phenomenon during the pandemic than before the pandemic. Only 1.85% believe that there are fewer violent behaviours in a family during the pandemic, while 39.35% state that this problem is currently present to the same extent as before the pandemic.

Furthermore, the number of aggressive behaviours of wards towards household members increased. It is stated by every second respondent (49.07%), and 46.75% of studied custodians assess the occurrence of this problem as the same as before the pandemic. Only 1.85% of custodians believe that this problem occurred more frequently before the pandemic. The obtained distribution of the frequency of answers is significantly different statistically (chi ^2^=94.02; df=2; p<0.05). On the one hand, pandemic conditions forced bigger social distance in the public space, but at the same time resulted in family members spending more time at home in small spaces, which forced more interactions that have not always been comfortable.

The increase in aggressive behaviours in wards during the pandemic occurred not only towards the closest family members. Every third custodian (33.33%) observed an increase in aggressive behaviours of wards towards other persons they know and 56.01% assess this problem as remaining at the same level. Only 2.31% of respondents believe that the number of such behaviours dropped during the pandemic. Differences in the obtained distribution are statistically significant (chi ^2^=102.76; df=2; p<0.05).

The scale of the hate phenomenon cyberbullying has also increased during the pandemic. This means that clients of probation officers more often used aggressive and negative comments on the internet. It was observed by 41.66% of respondents, whereas 43.98% believe that this problem occurs as frequently as before the pandemic and does not differ from before the pandemic. Only 1.85% of persons stated that this phenomenon currently occurs less frequently than before the pandemic. Obtained results indicate the existence of significant differences in the distribution of the frequency of answers (chi ^2^=83.08; df=2; p<0.05).

Additionally, custodians’ answers imply that 40.27% believe that their wards spend free time in a risky manner more frequently than in the period before the pandemic and 49.07% believe that their wards spent time in a risky manner before the pandemic and continue to do so. Only 5.09% of respondents believe that wards currently practice social distancing during their free time than before the pandemic. The distribution of obtained answers indicates the presence of statistically significant differences (chi ^2^= 74.32; df=2; p<0.05).

As many as 69.44% of court-appointed custodians observed an increase in the mental overload experienced by the loved ones of their wards. Perhaps this overload was caused by external restrictions resulting from the introduction of the pandemic, as well as growing interpersonal, family or personal problems resulting from the complications of restrictions. According to 25.04% of custodians the mental overload of their wards occurred before the pandemic and currently occurs with the same frequency. While 2.31% of custodians believe that the pandemic is favourable for the families of their wards and mental overload in these families occurred more frequently before the pandemic than currently. The obtained distribution of the frequency of answers indicates statistically significant differences (chi ^2^=156.18; df=2; p<0.05).

In summary, it can be seen that during the family situation during the pandemic, according to custodians participating in the research, problems with wards’ participation in school were growing the most (74.53%). The next position was taken by the mental overload of the ward’s loved ones (69.44%). Family conflict (67.12%) and domestic violence (56.94%) were ranked lower. Another place was taken by professional passiveness (55.55%) and worsening financial situation of families (54.62%). Another group of difficulties encountered by wards’ families relates to aggressive and risky behaviours, as well as alcohol abuse. These behaviours have increased in the opinion of approximately 40% of respondents. According to 37.5% of studied custodians, during the pandemic the frequency of reporting complaints by the family to the custodian with regard to the improper behaviour of the ward increased.

One-quarter of respondents (26.85%) believe that, during the pandemic, there was an increase in the incidence of wards being referred to residential educational care and rehabilitation facilities.

## Discussion

These results show that the COVID-19 pandemic resulted in a lot of changes among families under the supervision of a court-appointed custodian and a number of difficulties faced by the custodians occurred. Social problems that were disclosed during the COVID-19 pandemic are related to the prolonged exposure to worrying information, great uncertainty and life and health threatening conditions which cause various types of losses ( Hamer and Baran, 2021). Almost eight in ten adults identified the coronavirus pandemic as a significant source of stress in their lives ( Gruber *et al.*, 2021). Epidemic and related sanitary requirements cause problems in various spheres of human life: social, economic, cultural, as well as the education of children, youth and adults.

During the pandemic, on the grounds of the conducted research, an increase in the consumption of alcohol and intoxicants by court-appointed custodians’ wards and their family members has increased. This was a way to ease the tension easily and quickly ( Al’Absi, 2010).

Furthermore, we observe an escalation in the number of family conflicts, aggressive behaviours and domestic violence due to the lack of ability to solve conflicts through dialogue and negotiations. It may be assumed that the escalation of the violence is additionally affected by: isolation of persons in small housing facilities, drug and alcohol abuse, a deteriorating financial situation, intensification of problems that were present before the pandemic, lingering fear and living in stress, and untreated mental disorders. As indicated by the results of the presented research, during the pandemic the custodians’ wards usually remained under the influence of other demoralised persons, experienced professional inactivity, avoided participating in school classes and thus, have had more time and have remained outside of the social control. Such circumstances favour demoralisation and the occurrence of aggressive behaviours ( Urban, 2000).

Results of the survey indicate the growing phenomenon of cyberbullying which is increased by remote education, social distance and remote work ( Ahmed and Braithwaite, 2004). As a result of a lack of direct contact with other persons, interpersonal communication is transferred online and is reflected in social media. In the case of mediated communication, there is less sense of a partner’s presence in the communication ( Pyżalski, 2012). This leads to less focus on the person who we communicate with and mutual relations with this person. Additionally, perpetrators of online aggression usually feel anonymous which favours the occurrence of the phenomenon of disinhibition ( Aronson, 2005).

Moreover, we observe a decrease in motivation among custodians’ wards and their families to participate in educational classes and gainful employment. In the educational area the initial fascination with remote education resulting from the relatively easy way to obtain positive grades, may be reduced in ambitious children. Whereas children with specific needs have been to a large extent deprived of rehabilitation classes, skills training or early development support. Due to various reasons, many parents are not able to help their children with assignments, which is why they are left alone in this area as well.

The constantly changing situation makes it easier for children and youth to assume the following: “what is the point in trying and learning, if no one knows what will happen next”. They experience promises of return to schools and then, it turns out it is not possible. They notice that adults do not know what will happen. In effect, it may be assumed that some students log in remote classes and have no motivation to actively participate in them or do not log in at all.

Usually, every difficult situation brings some benefits. Online school education is comfortable for students characterised with a deficit in motivation. They do not have to commute to school. Consequently, these students may find it difficult to return to school, since they have developed strategies of functioning outside of the school. They can avoid stressful or uncomfortable situations which they encountered in school by using technical difficulties as excuses.

Whereas, as a result of stopping many interrelated industries, a lot of people lost their jobs or experienced a forced break in professional activity, which significantly affected the economic situation of many families ( GUS, 2020: 1). In a short time, the employee market, in which we observed many job offers, changed into the employer market ( GUS, 2020: 1). Thus, currently an employee has to make an effort to be employed and an employer does not strive for employment. In such a situation, court-appointed custodians’ wards, who are usually characterised with deficits in professional and personal competences, belong to the group who will be, among others, affected by the consequences of this change. The longer the pandemic lasts, the bigger economic diversification we can expect, where persons with high qualifications, intellectual and adaptive potential, who are technologically adept will adjust easier to new challenges.

Remaining in the conditions of the pandemic and staying with family members in small spaces deteriorated the functioning not only of custodians’ wards but also their families. Personal, family and interpersonal problems of wards under supervision of court-appointed custodians, which have been growing during the pandemic, lead to the intensified mental overload of their family members. Despite the implementation of a series of procedures and instructions for the period of the pandemic by the court guardianship, we cannot forget that each form of social isolation can lead to the occurrence of emotional, social and cognitive disorders ( Pratt, 2009). At the same time, the pandemic limited access to health care and psychotherapy for persons in these risk groups. Thus, in some cases support provided by the court-appointed custodian could have been the only available form of help.

### Limitations

One strength of the current study was its large sample, which included probation officers from all over Poland, specializing both in adult as well as in family and juvenile cases. This allowed for capturing the specifics of supervising clients of probation officers during the pandemic. However, the current study also has some limitations. One is the fact that it involved only probation officers in Poland. This has already been addressed in an ongoing research project seeking to include probation officers from other countries. Another limitation comes from the method of collecting data via the internet. The link with the questionnaire was sent to the business e-mails of probation officers but they could misplace the e-mail in their spam inbox, for instance. Conducting internet research may have contributed to the fact that the respondents were mainly people familiar with new technology.

Another limitation could be the lack of in-depth interviews with the probation officers. Such data would allow for a more detailed description of situations which probation officers find especially difficult during the pandemic. This suggests that further studies on probation officers using both qualitative and quantitative measures are warranted.

## Conclusions

The pandemic has changed social reality. It has changed the economy, revolutionised social systems, including the work of the administration of justice and court-appointed custodians. For persons easily influenced by negative information on social media, the pandemic is a period which intensified problems experienced by these persons, the feeling of uncertainty, fear and sometimes introduced loss and pain for their loved ones.

Therefore, it seems justified to provide quick support to families regarding skills in solving conflicts through mediation, communication training, financial education and access to free-of-charge psychotherapeutic support. For court-appointed custodians the introduction of education among wards regarding availability of institutions, organisations and persons providing the aforementioned support, is an important task.

Since we can expect further increase in the frequency of occurring personal and social problems experienced by court-appointed custodians’ wards, financial resources and energy of social institutions, cooperation with court guardianship should be focused on psycho-educational campaigns concentrating on providing information on symptoms of problems in the mental and emotional sphere. Preventing exclusion of groups at risk in the labour market and supporting families to prevent the escalation of psychopathology, development of addictions and domestic violence should be priority areas.

Furthermore, in order to extend the support provided for wards under the supervision of court-appointed custodians and unburden the court guardianship from the excess of cases, it would be valuable to create support systems in schools and social institutions by providing helplines, online duty hours and consultations. It is also necessary to develop psychoeducational programmes, psychological support, offers of environmental clubrooms, socio-therapeutic centres, mental health and pedagogical-psychological clinics with pandemic-related aspects.

## Data availability

### Underlying data

Figshare: data upload.csv. https://doi.org/10.6084/m9.figshare.19447193.v3 ( Opora, 2022).

This project contains the following underlying data:

- Data.xlsx [raw data collected during survey. The columns contain answers and the rows contain cases]

### Extended data

This project contains the following extended data:

- questionnaire in English.docx
- questionnaire in Polish.docx

Data are available under the terms of the Creative Commons Zero “No rights reserved” data waiver (CC0 1.0 Public domain dedication).
