# Supplementary material for: The family situation of wards under the supervision of court - appointed custodians during COVID-19 pandemic in Poland: a self-report study to examine the perception of probation officers
Source: F1000Res. 2022 Dec 19;11:1528. [Version 1] doi: 10.12688/f1000research.110435.1 (PMC9813447; doi:10.12688/f1000research.110435.1)
Supplement: Peer review history

# Peer review: Reviewer response for version 1

**DOI:** 10.5256/f1000research.122042.r158435

**Published:** 2023-01-05

**Authors:** Mary Grace Vella

**Affiliations:** 1 Department of Criminology, Faculty for Social Well-being, University of Malta, Msida, Malta

The article aimed to gain a better understanding of how the difficulties faced by clients of the Polish Probation Service have changed during the COVID-19 pandemic, and how this as a result has impacted the nature of work of probation officers. Thus, the aim of the study was to gain further insight into the views and perceptions of probation officers on the challenges encountered in the performance of their duties, with the aim of contributing towards the development of priority foci for future interventions and service provision post-Covid.  

 The application of this task was carried out through the adoption of a quantitative survey research by the use of a closed-ended questionnaire. The survey, conducted with 12 randomly selected court regions in Poland among 216 professional court-appointed custodians was sent to all probation officers operating in these regions. This research design was observed to offer a suitable and viable methodological framework for addressing the aims and purposes of the study and for gaining an overview of the transformations shaping the nature of work of Polish probation officers as a result of the pandemic. Due attempt has also been made to secure a cross-sectional representation of the probation workforce through the inclusion and participation of different court regions, to enable generalizability from the results. The study managed to achieve a significantly sized response rate, as from a total of 482 probation officers who received the questionnaire, 216 successfully submitted their responses. 

 The first part of the article aptly introduces the aims and objectives of the study and its underlying rationale, through embedding the research problem within the Covid-19 pandemic contextual framework.  The chapter demonstrates that the researcher has given ample initial reflection to the nature and purpose of the study, as well as to the overall methodological design and research process. Attention has also been addressed to the development of a suitable research question to focus and guide the conduction of the study. The underlying aim of the study is indeed adequately articulated through a relevant research question, i.e. ‘Have social problems in families under the supervision of a court-appointed custodian intensified during the pandemic?’ However, the paper could have presented a more detailed contextualized exploration of the researched population, by referring to the nature and structure of probation work in Poland, including the legislative framework governing community-based sanctions, the main duties and specialisations, caseload, as well as the main challenges associated with probation work. Such information is particularly relevant for those who are not familiar with the nature and structure of probation work in Poland, and would have provided a more comprehensive analysis of how court appointed supervision work operated pre-pandemic, thus enabling better comparability with the results emerging from the study.

 The methodology section of the study provides a succinct, yet inclusive overview of the methodological design of the study, including processes of data collection and analysis, as well as ethical considerations adopted for the purpose of the research.  The paper demonstrates insight of the methodological strengths and weaknesses of the study and has aptly attempted to mitigate these limitations and biases. A main strength of the study concerns the researcher’s successful attempt to gain response from a large sample of probation officers from all over Poland, specializing in juvenile, adult, as well as family cases. A methodological challenge which could have been further explored, concerns attempt to gain a more representative sample of probation officers in terms of socio-demographic characteristics and expertise, such as through the implementation of a clustered random sampling technique. Complementing the quantitative analysis through rich and in-depth qualitative data arising from interviewing with practicing probation officers would have enabled a more insightful and experiential approach, as well as a more holistic analysis of the issue under investigation. Despite the objective quantitative nature of the study which enables generalisability, recognition could be further made that given the examination of probation officers’ perceptions and experiences, the study is still based on the collation and measurement of subjective responses. The article has aptly put forward relevant recommendations and proposals for further research on the subject matter, including the location of the study within an ongoing wider comparative research project with probation officers from other countries. The article also provides a good overview of the ethical considerations taken on board to mitigate any arising ethical challenges arising from the conduction of the study, which given the anonymous nature of the survey research, were minimal.

 The findings arising from the survey research with probation officers have been presented in a systematic and orderly manner in both descriptive and tabular form. However, the findings could have highlighted a bit further the divergences, possibly through a compare and contrast approach, between pre- and post-pandemic experiences. The findings of the study were also aptly discussed and analyzed in line with existing contemporary literature in the field. Thus, the article confers a thorough and focused presentation and analysis of the findings as emerging from the results of the survey research.  

 The study pursues an interesting and highly relevant topic of analysis for the area of probation work. The research is well-designed and organised and the results arising from the study are aptly discussed and presented in line with important areas arising from the literature. The research has provided added contribution to knowledge not only in terms of the impact of the pandemic on social and probation work in Poland, but also in terms of the wider impact of the pandemic on multidimensional aspects of wellbeing. It is interesting to note that as in various other contexts, such as in the areas of social work and pedagogy, reduced caseload and restrictions posed by the pandemic, did not lead to a reduction in work for probation officers, but the complexity created by the pandemic situation instead led to the exploration for other alternative and innovative solutions.  

 On the basis of the research findings, the study has put forward a number of policy recommendations for addressing emerging trends and the amelioration of existing practices. Despite the validity of the proposed priority areas, referring amongst others to; holistic support to families, the consolidation of psycho-educational campaigns, addressing labour market exclusion for at-risk groups, as well as the development of support systems in educational and social institutions as well as various other services and programmes focusing on psychological health and wellbeing, proposals could have been more specifically focussed on addressing the particular challenges of court supervision and probation work. Through such concerted focus on the challenges facing probation work, by for example making reference to the need, if required, for legal reform and underlying philosophy or organisational structure and resources, would have provided more relevant contribution to knowledge by addressing the challenges of probation work in a more holistic and comprehensive manner.

Is the work clearly and accurately presented and does it cite the current literature?

Yes

If applicable, is the statistical analysis and its interpretation appropriate?

Yes

Are all the source data underlying the results available to ensure full reproducibility?

Yes

Is the study design appropriate and is the work technically sound?

Yes

Are the conclusions drawn adequately supported by the results?

Yes

Are sufficient details of methods and analysis provided to allow replication by others?

Yes

Reviewer Expertise:

Criminology, Restorative Justice,  Social Policy

I confirm that I have read this submission and believe that I have an appropriate level of expertise to confirm that it is of an acceptable scientific standard.

# Peer review: Reviewer response for version 1

**DOI:** 10.5256/f1000research.122042.r158436

**Published:** 2023-01-04

**Authors:** Barbara Contreras Montero

**Affiliations:** 1 Department of Sociology, Universidad de Granada, Granada, Spain

**General Comments**

 The article addresses an important issue regarding the perception of probation officers in Poland during COVID-19 about the family situation of wards under supervision. This is especially relevant from the perspective of Social Work, as long as it shows how the quarantine/isolation affected the families and the wards, and therefore the intervention of the probation officers.

 The strengths of this manuscript are:

1. There is a clear question or objective, which is measured by a number of relevant variables.
2. The methodology and sample are adequate.
3. In general, the data is well structured and well presented.
4. The discussion and conclusions show a relevant analysis of the topic.

 

**Specific Comments**

 However, some suggestions are given to improve the manuscript. reorder the document to make it more understandable. It is suggested:

- Review the introduction in order to detect some small errors in English or repetitions of the same word in the same sentence or the next one. For example, both, “video conference” and “videoconferencing” are admitted, but in the text is written “videoconferencing” an “video conferencing” Indistinctly. This needs consistency.
- In the seventh paragraph of the introduction, the first sentence "Describing the reality..." is catchable, since it does not provide relevant information and is not entirely linked to what follows.
- The analysis of the results is organized according to table 1. However, there is a small jump after the "narcotics/drugs use" and a disorganization in the last variables. It is understood that it is appropriate to comment on the section on “domestic violence” just before talking about other “aggressive behaviors”, but this loses the order of the table. It is recommended to rearrange the items in the table so that they correspond to the order of the analysis.
- There is a small typo/mistake in the comment about "Family fights". The table shows 0.46% while the analysis speaks of 0.45%. These numbers must match.

Recommendation: Approved with only minor changes.

Is the work clearly and accurately presented and does it cite the current literature?

Yes

If applicable, is the statistical analysis and its interpretation appropriate?

Yes

Are all the source data underlying the results available to ensure full reproducibility?

Yes

Is the study design appropriate and is the work technically sound?

Yes

Are the conclusions drawn adequately supported by the results?

Yes

Are sufficient details of methods and analysis provided to allow replication by others?

Yes

Reviewer Expertise:

Inequality and social exclusion, especially homelessness.

I confirm that I have read this submission and believe that I have an appropriate level of expertise to confirm that it is of an acceptable scientific standard.

# Peer review: Reviewer response for version 1

**DOI:** 10.5256/f1000research.122042.r158433

**Published:** 2023-01-03

**Authors:** Agnieszka Lewicka - Zelent

**Affiliations:** 1 Department of Social Rehabilitation Pedagogy, Uniwersytet Marii Curie-Skłodowskiej w Lublinie, Lublin, Poland

Despite the removal of restrictions in many countries, COVID-19 continues to take its deadly toll around the world, meaning that it is still present and threatening people. The author of the article has attempted to identify the problems faced by probation officers in Poland during the pandemic. Such findings are important in the context of the possibility of the return of sanitary restrictions resulting in changes in the ways in which probation officers do their work and support their charges. More than 200 probation officers participated in the author's research. Based on the results of the research, recommendations can be made for changes in the preparation of probation officers for their work and in the training of active probation officers. It would be worthwhile to see whether probation officers in other countries need to learn to cope with new difficulties, the effects of COVID-19.

Is the work clearly and accurately presented and does it cite the current literature?

Yes

If applicable, is the statistical analysis and its interpretation appropriate?

Yes

Are all the source data underlying the results available to ensure full reproducibility?

Yes

Is the study design appropriate and is the work technically sound?

Yes

Are the conclusions drawn adequately supported by the results?

Yes

Are sufficient details of methods and analysis provided to allow replication by others?

Yes

Reviewer Expertise:

special pedagogy, criminology, social rehabilitation, mediation,

I confirm that I have read this submission and believe that I have an appropriate level of expertise to confirm that it is of an acceptable scientific standard.
